# Algal and Cyanobacteria Cell Walls as Biosorbents for Phenolic Compounds: Comparative Performance and Sustainability Assessment of *Limnospira platensis *

**DOI:** 10.3390/bioengineering13040373

**Published:** 2026-03-24

**Authors:** Lorenzo Mollo, Alessandra Norici, Linda Raffaelli, Alessia Amato

**Affiliations:** 1 Enereco S.p.A., Via Einaudi 84/88, 61032 Fano, PU, Italy; l.mollo@staff.univpm.it; 2 Dipartimento di Scienze della Vita e dell’Ambiente, Università Politecnica delle Marche, 60131 Ancona, AN, Italy; linda.raffaelli9@gmail.com (L.R.); a.amato@univpm.it (A.A.); 3 Consorzio Interuniversitario Reattività Chimica e Catalisi (CIRCC), Via Celso Ulpiani, 27, 70126 Bari, BA, Italy

**Keywords:** microalgae, cyanobacteria, biosorption, LCA, cell wall, wastewater

## Abstract

Adsorption is a method widely used to remove low-molecular-weight organics from wastewaters, and phenolic compounds from olive mill wastewater are a persistent class of bioactive pollutants of environmental concern. We screened eleven microalgal candidates at 0.10 g·L^−1^ using batch kinetics fitted with the Lagergren pseudo-first-order model to obtain rate constants (k) and fitted equilibrium capacities (q_e_). Cyanobacteria, particularly *Anabaena* spp. and *Limnospira platensis*, exhibited the highest adsorptive potential in the screening; wall-less species (e.g., *Dunaliella salina*, *Isochrysis galbana*) showed negligible surface adsorption, indicating that the presence and type of cell wall highly influence biosorption. *L. platensis* was selected for detailed study because of its established industrial cultivation and valorisation potential. Equilibrium experiments with HCl-functionalized *L. platensis* at four biomass loadings (0.10–1.00 g·L^−1^; initial phenolic mix 30 mg·L^−1^) showed that increasing dose reduced equilibrium concentration (C_e_) but decreased specific uptake from ≈77 mg·g^−1^ to ≈18 mg·g^−1^ while removal rose from ~26% to ~61%. Nonlinear isotherm fitting favoured the Freundlich model (1/n < 1), consistent with heterogeneous, multi-site adsorption. Targeted macromolecular extractions abolished phenol uptake, demonstrating that the intact protein–polysaccharide matrix is essential for binding. *L. platensis* route delivered higher single-cycle removal (≈61%) compared to the maize-derived activated carbon reference (≈49%) while also incurring a 1.3-fold lower GWP (approximately 3 kg CO_2_-eq per treatment) than the activated carbon route (approximately 4 kg CO_2_-eq per treatment) in our model. Overall, *L. platensis* represents a lower-impact alternative for natural phenols remediation, especially when integrated into valorisation pathways that recover algal co-products.

## 1. Introduction

Olive mill wastewaters (OMWWs) are high-volume liquid residues generated during olive oil extraction. Their production is concentrated in the Mediterranean basin, where olive oil is a key agro-industrial sector [1]. Italy, Spain, and Greece together account for more than two-thirds of global olive oil production, and the associated OMWW streams reach tens of millions of cubic metres annually, with sharp seasonal peaks [2,3]. The scale and intensity of these effluents make their sustainable management a pressing regional challenge.

OMWWs are acidic effluents characterized by very high chemical oxygen demand (COD), suspended solids, salts, and, most critically, exceptionally high concentrations of phenolic compounds [3,4]. These phenolics, which can occur at gram-per-litre levels, are of interest for nutraceutical applications when selectively recovered [5,6] but are otherwise toxic and persistent pollutants. Their antimicrobial and phytotoxic properties inhibit soil microbial activity, reduce plant growth, and cause acute aquatic toxicity when OMWWs are discharged untreated or used in uncontrolled land-spreading [7,8,9].

The management of OMWWs in Italy and Europe currently involves agronomic land application under strict regulations, physic-chemical treatments (adsorption, coagulation–flocculation, membrane filtration), biological processes (aerobic and anaerobic digestion), and advanced oxidation for refractory organics [10,11]. Each approach faces limitations: adsorption and solvent extraction are costly, biological treatments are hampered by the inhibitory effect of phenolics, and oxidative processes are energy- and reagent-intensive. Integrated treatment strategies combining physical, chemical and biological processes are increasingly advocated for OMWW, but they are often constrained by high operational costs, scale-up challenges, and effluent quality limitations [12,13,14].

In this context, microalgae and cyanobacteria are gaining attention as potential biotechnological tools for OMWW treatment [15,16]. These biological systems offer a dual function: they can assimilate nutrients and some organic matter while producing biomass that can be valorised in biorefinery chains [17,18]. Moreover, both live and non-living biomass can act as biosorbents, binding pollutants through abundant surface functional groups such as carboxyl, hydroxyl, amine, and phosphate moieties [19,20,21,22]. Beyond environmental remediation, coproducts such as proteins, lipids, carbohydrates, and pigments (e.g., phycocyanin) provide opportunities to offset treatment costs and improve circularity [23,24].

Biosorption by algal biomass has been demonstrated across diverse pollutant classes, including phenolic compounds, pharmaceuticals, dyes, and heavy metals such as Pb(II), Cd(II), and Cu(II) [21,22,25]. Removal efficiencies are frequently comparable to those of conventional sorbents, and biomass versatility allows adaptation to different matrices. Mechanistically, adsorption involves a combination of hydrogen bonding, electrostatic attraction, hydrophobic interactions, and complexation with cell-surface polymers and extracellular substances [26]. Notably, studies report that dried or chemically modified biomass often exhibits higher adsorption stability than live cultures, and performance is strongly influenced by solution chemistry (pH, salinity, ionic strength) [27,28,29,30], conditions particularly relevant for the acidic, saline character of OMWWs.

Evidence highlights the versatility of algal sorbents across pollutants: pharmaceuticals, heavy metals and phenolic compounds are among the main contaminants for which algae have been used for removal [31,32,33,34,35,36,37]. This breadth suggests multiple deployment options in OMWW treatment, such as a primary adsorptive step targeting phenolics or a polishing stage integrated into existing treatment trains. Importantly, the biomass generated could be further processed, extending its use in cascaded biorefinery schemes.

Not all algae, however, exhibit equivalent adsorption performance. The polyphyletic nature of microalgae means wide structural diversity in cell-wall architecture and surface chemistry [38,39,40,41]. Chlorophytes typically have cellulose-rich walls [42], diatoms incorporate silica frustules [43], cyanobacteria feature peptidoglycan-based cell walls and, in some species, proteinaceous sheaths [44]. Nonetheless, some taxa such as *Dunaliella* lack rigid cell walls entirely [45]. These differences dictate porosity, functional group abundance, and accessibility of binding sites, resulting in marked species-specific adsorption behaviour. It should be noted that integrating compound extraction and biomass valorisation into a biorefinery workflow could influence biosorption performance: depending on the extraction method and the target coproducts, adsorption may decrease, remain unchanged, or even improve [46,47,48].

Sustainability is central to evaluating algal-based OMWW treatments. While algal biosorbents can reduce reliance on energy-intensive materials like activated carbon, their net environmental benefit depends on optimizing the cultivation and processing stages. In this framework, phycoremediation sustainability hinges on the synergy between adsorption performance and biological productivity. Identifying species that combine high pollutant uptake with industrial scalability is vital to ensure that the environmental gains are not offset by the resources required for biomass production. Crucially, unlike traditional sorbents, which require high-temperature carbonization and aggressive chemical activation, microalgae represent a regenerative, carbon-negative pathway. By fixing atmospheric CO_2_ during growth, they transform remediation from a simple waste-management task into a pillar of industrial decarbonization. Life Cycle Assessment (LCA) is therefore essential to quantify these trade-offs and benchmark algal biosorption against conventional technologies [49,50,51].

The aim of this study is threefold: (i) to screen the phenolic adsorption capacity of different microalgal and cyanobacterial species to identify candidates that offer both high removal efficiency and industrial feasibility, explicitly comparing wall-bearing and wall-less strains to confirm the active role of the cell envelope; (ii) to evaluate how the prior extraction of high-value macromolecules affects the subsequent adsorption capacity of a species selected for its optimal balance between adsorption potential, biological productivity, and established industrial relevance, thus simulating a cascading biorefinery approach; and (iii) to assess the environmental performance of algal biosorption relative to conventional activated carbon treatment through a comparative LCA. By combining species-level screening, mechanistic evaluation, and sustainability analysis, this work seeks to clarify both the feasibility and environmental implications of integrating algal biosorbents into OMWW management strategies.

## 2. Materials and Methods

### 2.1. Algal Cultivation and Biomass Functionalisation

This research included 11 microalgal and cyanobacterial species selected to represent a wide diversity of cell wall architectures. To evaluate the influence of parietal composition on adsorption, the panel comprised species with cellulosic walls (Chlorophytes), siliceous frustules (Diatoms), and peptidoglycan-based envelopes (Cyanobacteria), as well as species lacking a rigid cell wall (e.g., *Dunaliella salina*) included as reference models for surface interaction mechanisms. All the species, together with their phylum, origin (freshwater or marine environment), ID code, and type of cell wall are reported in Table 1.

Cultures were grown in batch flasks under continuous illumination (100 µE·m^−2^·s^1^) at 20 °C. Cells were harvested during the late exponential phase by centrifugation (3500 rpm 10 min) and washed two times with deionized water to remove any possible residues of the growth medium. The wet biomass was then functionalised by suspending it in a solution of HCl 0.1 M for 20 min [52]. The functionalisation allowed the release of the binding site on the algal cell wall [52]. After the functionalisation, the HCl was removed by centrifugation, and the biomass was dried at 80 °C for at least 24 h or until a constant weight was achieved. Then, the dry biomass was manually ground to a fine powder using a pestle. To ensure experimental reproducibility and minimize variations in surface area, the grinding process strictly followed the standardized protocol reported by Pennesi et al. [52], achieving a visually uniform consistency across all samples.

### 2.2. Limnospira Platensis Growth and Characterization

Among the screened species, *L. platensis* was selected for detailed investigation not only for its adsorption potential but primarily due to its established industrial relevance and suitability for cascading valorization. This cyanobacterium represents an ideal candidate for a biorefinery approach, where high-value compounds can be recovered before using the residual biomass for wastewater treatment. The species was grown in triplicate in 250 mL flasks filled with 100 mL of Zarrouk medium [53] for 15 days (reaching of the stationary phase). The cultures were grown under continuous illumination (100 µE·m^−2^·s^−1^) at 20 °C. The growth of *L. platensis* was monitored spectrophotometrically by measuring the optical density at 670 nm [54]. A calibration curve was established by correlating OD_670_ values with dry weight, which was determined from samples previously analyzed spectrophotometrically, subsequently washed with distilled water to remove residual medium salts, and dried to constant weight. This calibration was then applied to construct a growth curve expressed as dry weight (g L^−1^) over time (days).

Biomass of *L. platensis* was tested after extraction of phycobiliproteins and soluble proteins. The remaining biomass was then washed, functionalised and dried as previously reported. Phycobiliproteins were extracted by adding 1 mL of phosphate buffer (0.05 M, pH 6.7) containing lysozyme (10 mM) [55], and extraction efficiency was enhanced by mechanical cell disruption using high-pressure nitrogen (cell disruption bomb, Parr Instruments). The extraction was carried out at 4 °C for 24 h, and the supernatant containing the pigments was collected after centrifugation at 10,000 rpm for 15 min. Phycocyanin (PC), allophycocyanin (APC), and phycoerythrin (PE) were quantified spectrophotometrically (UV-1900i, SHIMADZU CORP., Kyoto, Japan) at 562, 615, and 652 nm using quartz cuvettes, and concentrations (mg L^−1^) were calculated according to Singh et al. [56].

The total protein content of *L. platensis* in stationary phase was determined following the Lowry method with modifications introduced by Peterson [57]. Briefly, 2 mL of culture were centrifuged (2000 rpm, 10 min), and the pellet was resuspended in 500 µL of 1% (*w*/*v*) SDS with 0.1 M NaOH. After vortexing, 500 µL of reagent A were added, followed after 10 min by 250 µL of reagent B. Samples were incubated for 30 min to allow colour development. Protein quantification was performed spectrophotometrically at 750 nm (UV-1900i, SHIMADZU CORP.) using a calibration curve previously obtained with bovine serum albumin (BSA, 0–90 µg µL^−1^).

### 2.3. Phenol Adsorption Experiments

Phenol adsorption was investigated through batch equilibrium assays. A stock solution (1 g·L^−1^) was prepared containing three phenolic compounds: caffeic acid, tyrosol, and *p*-coumaric acid [54]. The phenolic compounds were selected based on their reported concentrations in olive mill wastewaters by Deeb et al. [58]. The compounds were purchased from Sigma-Aldrich Corporation: tyrosol (C_8_H_10_O_2_, MW 138.16 g·mol^−1^, purity 98%, HPLC), p-coumaric acid (C_9_H_8_O_3_, MW 164.05 g·mol^−1^, purity ≥98%, HPLC), and caffeic acid (C_9_H_8_O_4_, MW 180.16 g·mol^−1^, purity ≥ 98%, HPLC). Working solutions were freshly prepared by dilution with distilled water to the desired initial concentrations.

Batch adsorption tests were conducted in 15 mL tubes (10 mL working volume) or 250 mL Erlenmeyer flasks (100 mL working volume), containing the phenolic solution and a defined mass of algal biomass. The suspensions were agitated at 100 rpm at 25 °C. Contact times ranged from 5 to 60 min for kinetic assays and up to 24 h for equilibrium studies.

At predetermined time intervals (t = 0, 5, 10, 15, 20, 30, 45, 60 min), 1 mL aliquots were withdrawn, centrifuged at 3000 rpm for a few seconds, and the supernatant analyzed for residual phenolics using a modified version of the Folin–Ciocalteu method [54,59]. Briefly, 125 µL of sample were mixed with 125 µL of Folin reagent and 500 µL distilled water, vortexed and allowed to react for 6 min. Then, 2.25 mL of NaHCO_3_ (5.56%) were added, and samples were incubated in the dark for 90 min. Absorbance was measured at 760 nm with a UV–Vis spectrophotometer (UV-1900i, SHIMADZU CORP.) using quartz cuvettes. Phenolic concentration was quantified against a calibration curve prepared with gallic acid, and results are expressed as gallic acid equivalents (mgEq GA·L^−1^).

Two sets of adsorption experiments were performed:

• Species screening—A fixed biomass dose of 0.1 g·L^−1^ (dry weight) was tested for all algal species with phenolic solution (30 mg·L^−1^) to compare uptake capacity.
• Dose optimization with *L. platensis*—Biomass concentration was varied (0.1–1.0 g·L^−1^) with 30 mg·L^−1^ phenolic solution to evaluate adsorption as a function of biomass-to-phenol ratio. Untreated, pigment-extracted, and protein-extracted *L. platensis* were tested under identical conditions.

All adsorption tests were performed in triplicate.

#### Fitting of Adsorption Data

To select the most performant biosorbent for subsequent isotherm and optimization experiments, adsorption kinetics measured at a fixed biomass loading (0.10 g·L^−1^) and an initial phenolic concentration of 30 mg·L^−1^ were analyzed using the Lagergren pseudo-first-order model [60] expressed in differential form as

$$\frac{{dq}_{t}}{dt}=k\;(q_{e}-q_{t})$$

where *q_t_* (mg·g^−1^) is the amount adsorbed at time *t*, *q_e_* (mg·g^−1^) is the equilibrium uptake and *k* is the pseudo-first-order rate constant [33]. In this study k is expressed in ms^−1^. For each species, k and q_e_ were obtained by nonlinear least-squares fitting of the time-series data. The fitted rate constant k was interpreted as an indicator of adsorption speed, while the fitted equilibrium uptake q_e_ was used as the practical comparative metric to rank species performance and select the optimal biomass for the dose–response and isotherm investigations.

Equilibrium adsorption data obtained from dose-optimization experiments with *L. platensis* were analyzed by nonlinear least-squares regression using the Levenberg–Marquardt algorithm [61]. Two commonly used isotherm models were evaluated: the Langmuir model [62], which assumes monolayer adsorption onto a finite number of identical sites, and the Freundlich model [63], an empirical expression for heterogeneous surfaces. Model performance was assessed by R^2^ and RMSE, and the best-fitting model was used to inform mechanistic interpretation of the observed dependence of *q_e_* on biomass dose. The Langmuir model is given by

$$q_{e}=\frac{Q_{0}K\;C_{e}}{1+K\;C_{e}}$$

where *q_e_* (mg g^−1^) is the adsorbed amount at equilibrium, *C_e_* (mg L^−1^) is the equilibrium solute concentration, *Q*_0_ (mg g^−1^) is the maximum (monolayer) adsorption capacity, and *K* (L mg^−1^) is the Langmuir affinity constant. The Freundlich model was used in the form:

$$q_{e}=K_{f}\;C_{e}^{1/n}$$

where *K_f_* is the Freundlich constant related to adsorption capacity and *n* is a dimensionless heterogeneity (intensity) factor. Here, 1/*n* < 1 indicates a concave isotherm (favourable adsorption).

### 2.4. Life Cycle Assessment (LCA) Methodology

The LCA tool was used with to estimate the sustainability of the phycoremediation approach using *L. platensis* (Scenario 1), compared with the most common adsorption onto activated carbon (Scenario 2). The LCA study was performed in accordance with UNI EN ISO 14040 [64] and UNI EN ISO 14044 standards [65]. LCA for Experts software (v. 2025.2), integrated with the Life Cycle Engineering database, was used for data collection. The analysis focused on the impact category of climate change, expressed in kg CO_2_ equivalents [66]. This measure compares the global warming potential (GWP) of each greenhouse gas with that of carbon dioxide, which is assigned a reference value of 1.

The impact was estimated by the method Environmental Footprint 3.0 [67], in agreement with EC Recommendation 2013/179/EU. This method was applied in both the classification and characterization phases. The methodological framework followed consolidated LCA practices, consistent with previous applications of LCA to algal-based systems [68]. The analysis, including mass and energy balances, referred to a functional unit of 1 L of phenolic solution to treat.

**Figure 1:**
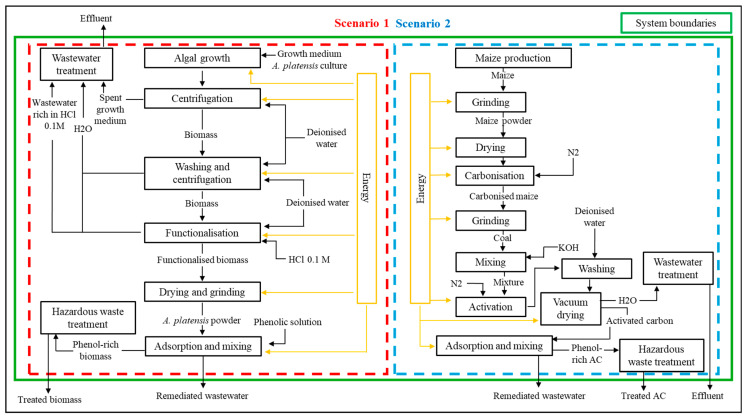
System boundaries of the two scenarios considered in the Life Cycle Assessment (LCA). Scenario 1: biosorption of phenols using functionalized Limnospira platensis biomass. Scenario 2: adsorption using activated carbon. Where AC is reported it refers to Activated Carbon.

System boundaries of the two scenarios considered in the Life Cycle Assessment (LCA) are reported in Figure 1. Activated carbon was chosen as a benchmark adsorbent for Scenario 2 due to its well-documented effectiveness for removing low-molecular-weight organics such as phenols [69,70]. Liquid-phase uptake on activated carbons depends on adsorbent properties (pore structure, ash content and surface functional groups) and on solution conditions (pKa, polarity, pH, ionic strength and solute concentration) [70]. Activated carbon is available in powdered, granular and fibrous formats that differ in mass-transfer behaviour; selection of a precursor typically favours low inorganic content, ease of activation, availability and low cost. For comparison we refer to maize-kernel-derived activated carbon [69] as a representative example meeting these criteria (reported surface areas >2000 m^2^·g^−1^), noting that this material was adopted only as a comparative reference and was not produced within the present work.

Some assumptions were necessary to ensure consistency between scenarios. The CO_2_-related impact derived from energy consumption was calculated based on the average European grid mix (0.3 kg CO_2_ kWh^−1^). Then, pre-treatment stages of the effluent for phenol concentration reduction were excluded, as they are common to both scenarios. It was assumed that biomass washing in both scenarios was carried out with distilled water, despite this being unspecified for Scenario 2. Furthermore, the two phenolic solutions were considered to have comparable concentrations, although Scenario 1 employed a concentration of 30 mg L^−1^ and Scenario 2 a concentration of 100 mg L^−1^. To compare the phenol adsorption capacity, equilibrium concentrations (q_e_) were correlated using the Langmuir isotherm.

Finally, the adsorption process was analyzed considering the influence of pH. According to Park et al. [69], phenol adsorption on activated carbon is maximized under acidic conditions (pH = 3). Their study varied pH from 3 to 12 while maintaining constant adsorbent dose, solution volume, and stirring rate, with pH adjusted using 0.1 M HCl and 0.1 M NaOH. This finding supports the assumption that initial acidic conditions favour adsorption efficiency. The treated effluent leaving the system boundaries was assumed to meet discharge standards and thus was considered ready for reuse or aquifer recharge, without additional post-treatment steps. At the end of the process, both the algal biomass and the activated carbon enriched in phenolic compounds were hypothetically treated as hazardous waste, applying an average EU treatment factor of 0.440 kg CO_2_eq per kg of waste to account for disposal impacts.

### 2.5. Statistical Analysis

Each adsorption experiment was conducted with a minimum of three independent replicates. *L. platensis* was grown in three independent biological replicas. Results are expressed as the mean accompanied by the standard deviation (±SD).

GraphPad Prism 9.5.0 (GraphPad Software) was used to perform statistical analysis. All statistical tests were carried out using a significance threshold of a = 0.05. One way ANOVA, followed by Tukey’s post hoc test, was used to compare adsorption values between each treatment. Letters were used in tables to distinguish significantly (*p* < 0.05) different treatment.

## 3. Results

### 3.1. Species Screening

**Figure 2:**
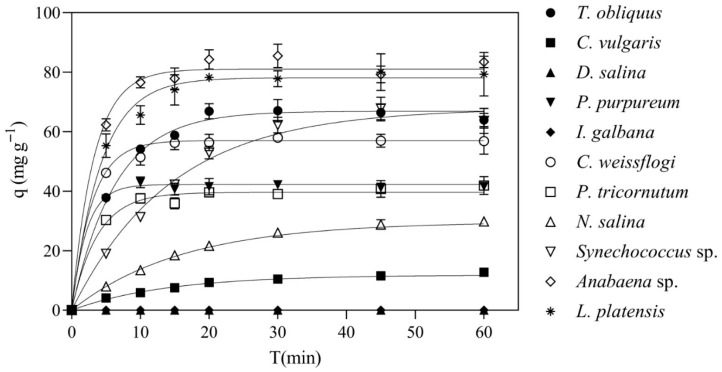
Comparison of phenolic adsorption capacities (q) over time across tested species. Each curve represents the average experimental trend. Experimental data are reported as mean ± standard deviation.

The adsorption performance of the candidate species is summarized in Table 2 and graphically reported in Figure 2. Under the screening conditions (0.1 g L^−1^ biomass, 30 mg L^−1^ initial phenol, 1 h contact), *Anabaena* sp. achieved the highest removal. Specifically, the amount of phenols adsorbed at equilibrium by the algae was 81.5 ± 7.8 mg·g^–1^. The other cyanobacteria also performed well, with average qₑ values above 65 mg g^−1^. In particular, *L. platensis* performed close to *Anabaena* sp. with a qₑ value of 76.9 ± 6.0 mg g^–1^. Among chlorophytes, *T. obliquus* showed adsorption comparable to *Synechococcus* sp. (66.6 ± 6.7 mg g^–1^), whereas *C. vulgaris* and *D. salina* exhibited much lower performance, with the latter showing no detectable adsorption. The rhodophyte *P. purpureum* and the bacillariophytes *C. weissflogi* and *P. tricornutum* showed intermediate performance between *Anabaena* sp. and *D. salina*. Finally, *N. salina* exhibited only limited adsorption (qₑ = 28.1 ± 8.0 mg g^–1^), while *I. galbana*, similar to *D. salina*, showed no measurable uptake.

Although apparent differences in the pseudo first-order rate constant of biosorption (k) were observed among some groups (Table 2), the one-way ANOVA did not reveal statistically significant differences (*p* = 0.124). This lack of significance is likely due to the high within-group variability together with the limited number of replicates (n = 3), which reduced the statistical power of the test.

### 3.2. Optimization of Limnospira Platensis as Biosorbent

**Figure 3:**
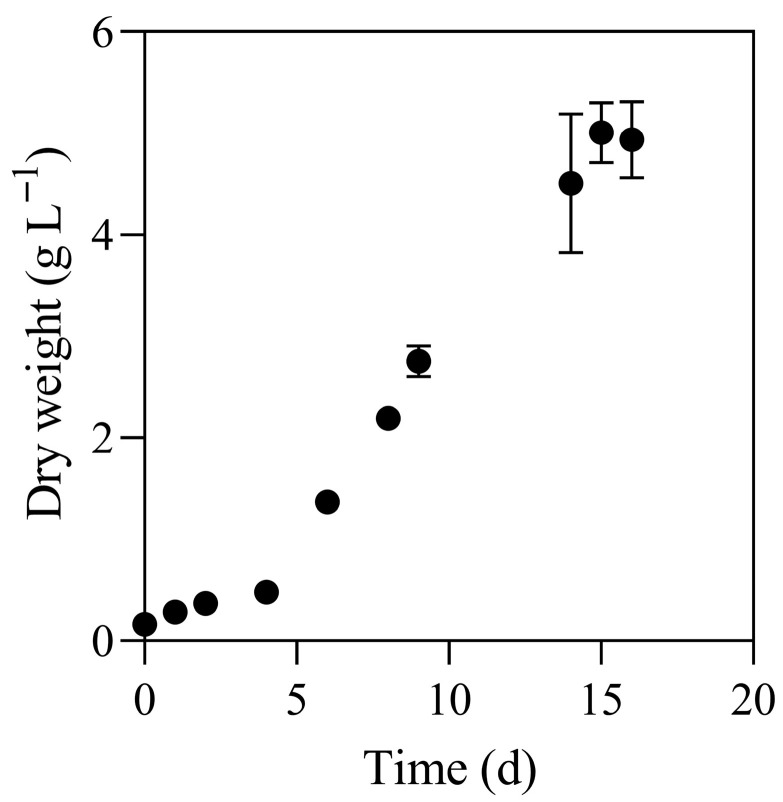
Growth curve of Limnospira platensis. Values are presented as mean ± standard deviation. The maximum biomass concentration (4.5 g·L^−1^) and the corresponding cultivation time required to reach the stationary phase (15 days) were considered as input parameters for the LCA analysis.

Batch adsorption data for functionalized *L. platensis* (Spirulina) exposed to a 30 mg L^−1^ phenolic mixture were collected at several biomass doses (0.1–1.0 g L^−1^). The data show the common “dose effect”: increasing adsorbent loading increases percent removal (lower C_e_), while the uptake normalized per unit mass (q_e_) decreases markedly (Table 3). For example, at 0.1 g L^−1^ the equilibrium adsorption was 76.9 ± 6.0 mg g^−1^, while at 1.0 g L^−1^ it fell to roughly 18 mg g^−1^. Correspondingly, the residual phenol in solution (C_e_) decreased from about 22 mg L^−1^ at low dose to ~12 mg L^−1^ at the highest dose, indicating higher total removal but lower uptake per gram at higher adsorbent loading. Figure 3 plots q_e_ versus *C*_e_ and shows that the adsorption curve has a steep initial slope and no clear plateau within this range.

Nonlinear fitting of these data produced the following isotherm parameters. The Langmuir model yielded an unreasonably large capacity Q_0_ of 1.07 × 10^7^ mg g^−1^ and an extremely small affinity *K* of 2.8 × 10^−7^ L mg^−1 ^(Table 4). These results indicate that a Langmuir monolayer plateau was not observed over the experimental concentration range: the system behaved nearly linearly within the measured domain and Langmuir is therefore not appropriate to derive a meaningful Q_max_.

By contrast, the Freundlich fit gave a K_f_ of 0.746 mg g^−1^ and a 1/n value of 1.55 (Table 4). The Freundlich model provides a superior and physically coherent description of the experimental data compared with Langmuir.

**Table 4:** Isotherm parameters for phenol adsorption onto *L. platensis*. Data were fitted using nonlinear regression on the experimental means from dose–response assays. Values are reported as mean ± standard deviation.

Model	Parameter	Value			R^2^
Langmuir	Q_max_ (mg g^−1^)	1.38 × 10^7^	±	2.4 × 10^6^	0.732
	K_L_ (L mg^−1^)	2.8 × 10^−7^	±	1.2 × 10^−7^	
Freundlich	K_F_ (mg g^−1^)	0.746	±	0.231	0.821
	1/n	1.55	±	0.59	

### 3.3. L. platensis as Biosorbent After Protein or Phycobilin Extraction

The phycobiliprotein analysis revealed that phycocyanin was the most abundant pigment, followed by allophycocyanin and phycoerythrin: the pigment content was respectively 17.6 ± 2.5%, 4.1 ± 0.3%, and 1.8 ± 0.4%.

Protein extraction, quantified through the Lowry–Peterson method, indicated a protein content of 55.3 ± 11.4%, consistent with the highly abundant protein fraction typically reported for *L. platensis*.

Regarding biosorption performance, the biomass previously subjected to phycobilin or protein extraction was tested, and no phenol adsorption was observed.

### 3.4. LCA Analysis of L. platensis as Biosorbent

A cradle-to-gate approach (from production to use) was used to determine the environmental impact of using *L. platensis* as biosorbent for OMWWs deriving phenols. Impact of algal cultivation was calculated based on growth data acquired from experimental analysis carried out in the laboratory. Algal growth was followed until it reached a stationary phase (day 15) where a biomass dry weight of 4.5 ± 0.2 g L^−1^ was obtained (Figure 3). Data from the growth curve (i.e., the biomass production, the consumption of growth medium and the energy demand) were used for LCA analysis, while the collected biomass was used for biosorption experiments and macromolecules extraction as reported in detail in Section 3.3.

Life cycle inventory data were then compiled for two treatment scenarios: adsorption of phenolic compounds using HCl-functionalized *Limnospira platensis* biomass, and adsorption using maize-derived activated carbon (Scenario 2). Inventory inputs considered energy use for biomass and adsorbent preparation, chemical reagents, water and wastewater treatment, and material flows for the adsorption stage (Table 5).

**Figure 4:**
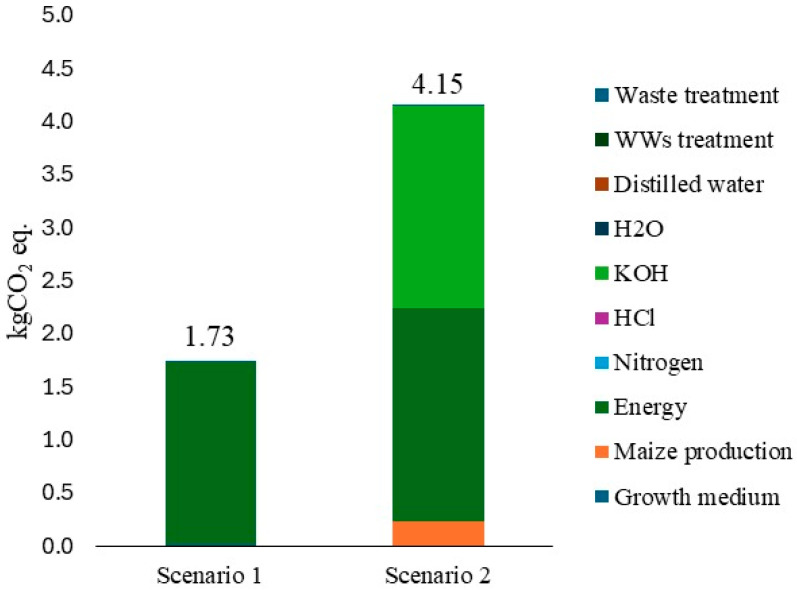
LCA contribution analysis of global warming potential (kg CO_2_-eq per treatment) for *Limnospira platensis* biosorption and maize-derived activated-carbon adsorption. Colours indicate process-level contributions (functional unit: 1 L of phenolic solution). Some categories included in the legend may not be visible in the bars because their relative contribution is negligible (<1%) compared to the main impact drivers.

The LCA results (Figure 4) indicate a clear divergence in climate impacts between the two scenarios: the activated carbon (AC) route (Scenario 2, 69) shows substantially higher greenhouse gas (GHG) emissions compared to the Spirulina biosorbent pathway (Scenario 1). On a per-functional-unit basis, the carbon footprint of Scenario 2 is several times larger than that of Scenario 1. This discrepancy is mainly due to the energy- and chemical-intensive steps required to produce activated carbon. In particular, KOH activation and high-temperature carbonization consume several kWh per batch, while additional milling and vacuum-drying stages add further electrical demand. The production and use of KOH itself also represent a significant burden, such that these two contributions, furnace electricity and alkali activation, account for the overwhelming majority of Scenario 2’s global warming potential (GWP).

In contrast, Scenario 1 consists of cultivation of *L. platensis* in Zarrouk medium, harvesting and drying of the biomass, and a mild functionalization step using HCl. The main impacts here originate from algal cultivation, primarily nutrient supply and mixing/harvesting energy, and, to a lesser extent, the acid functionalization stage. Nevertheless, the overall burdens remain considerably smaller than those of Scenario 2. An additional consideration is that autotrophic cultivation inherently fixes CO_2_ via photosynthesis. Large-scale LCAs of *L. platensis* cultivation have reported net CO_2_ sequestration values of approximately 0.70 kg CO_2_-eq per kg of dry biomass, after accounting for respiration losses and carbon uptake inefficiencies [71,72,73]. Although the theoretical fixation potential exceeds 1.5 kg CO_2_ kg, actual values are lower due to metabolic CO_2_ release and suboptimal conversion efficiencies, often ranging between 30 and 60. The present study did not explicitly account for CO_2_ uptake within system boundaries; this aspect highlights a potential for further reducing net emissions in algae-based processes relative to fossil-derived carbons.

Breaking down the contributions by process stage further emphasizes these differences (Figure 4). In Scenario 2, more than two-thirds of the total GWP derives from the combined energy demand of the activation furnaces and the chemical footprint of KOH production, with the remainder attributable to ancillary electricity for drying and milling. Notably, the impact associated with maize cultivation accounts for just over 5% of the total. By contrast, Scenario 1 presents a more homogeneous but still energy-driven profile: over 98% of the total GWP arises from energy consumption during cultivation, agitation, and harvesting, while the contribution from the growth medium accounts for approximately 1.3%. Other flows, such as water consumption during cultivation and functionalization or wastewater treatment at end-of-life, were modelled for both systems, but their impacts are marginal compared to energy and chemical use.

The comparative outcome is therefore robust: the biosorption scenario achieves treatment with an impact on the climate change on the order of only a few kg CO_2_-eq per unit of treated effluent (1.7 kg CO_2_eq), while the activated carbon route reaches values 2.4 times higher. This result underscores how the reliance on high-temperature, chemically intensive processing in conventional carbon production drives substantially larger burdens than the comparatively low-energy, biologically based *L. platensis* pathway.

Beyond climate impacts, the scenarios also differed in performance: in our experiments, the HCl-functionalized *L. platensis* biosorbent (Scenario 1) achieved the highest removal efficiency, with approximately 61% of the phenolic fraction eliminated in a single batch treatment. By contrast, the maize-derived AC (Scenario 2) removed about 49% under its test conditions. Thus, the Spirulina biosorbent not only carries a substantially smaller life cycle climate burden (roughly an order of magnitude less CO_2_-eq per functional unit) but also achieves greater removal performance.

## 4. Discussion

### 4.1. The Role of Algal Cell Wall in Adsorbing Phenols

The absence of a true cell wall has a direct and measurable consequence on phenol removal: species lacking a structured fibrillar wall (for example *Dunaliella salina* and *Isochrysis galbana* in our screening) show negligible surface adsorption of phenolic compounds under the conditions tested, a pattern that was observed in vivo by Mollo et al. [54]. Microalgae can remove dissolved pollutants by three partially overlapping mechanisms that together determine their remediation potential [74,75,76,77]. (1) Biosorption: a rapid, passive process in which contaminants are adsorbed onto exposed functional groups of the cell wall or extracellular matrix. This mechanism depends directly on the presence, composition and accessibility of wall-bound moieties (hydroxyl, carboxyl, sulphate, amine, aromatic residues) and is therefore severely impaired or absent in wall-less taxa [78,79]. (2) Bioaccumulation: it involves active or facilitated transport across the cell envelope and intracellular sequestration. Bioaccumulation requires intact membranes and living metabolism and can lead to accumulation of parent compounds or transformation products inside the cell [80,81]. (3) Biodegradation: it denotes enzymatic transformation or mineralization, either intracellularly or via extracellular enzymes, and it yields permanent removal only when metabolic pathways capable of degrading the target compound are present and active [82,83].

Because biosorption is the mechanism most dependent on an intact, chemically rich cell wall, its absence in species such as *D. salina* or *I. galbana* explains the largely absent surface uptake measured here; in such taxa overall removal therefore relies more heavily on bioaccumulation and biodegradation (if present), as reported by Mollo et al. [54]. This is further supported by the literature demonstrating the capacity of wall-less *Dunaliella* species to internalize and enzymatically degrade complex organic pollutants [84,85], or to rely on synergistic interactions with the microbiome. For instance, in the case of phenol, *D. salina* achieves significant removal when co-cultured with specific bacteria (e.g., *Halomonas* sp.), although the primary degradative metabolism in such consortia is predominantly driven by the bacterial partner [86]. In general, living algae tend to achieve higher overall removal rates than dried or inactivated biomass, since they can exploit the combined action of biosorption, bioaccumulation and biodegradation [54,87,88,89]. However, tolerance to phenolic compounds varies markedly across species, phyla and compound types, and in less tolerant taxa the metabolic contribution may be negligible or even impaired under realistic exposure scenarios. In these cases, dried biomass can occasionally perform comparably or even more efficiently than living cells, since the absence of active metabolism prevents toxic effects that would otherwise suppress uptake, a dynamic well-documented for algal biomass exposed to high concentrations of toxic organics [90,91].

The cell wall of algae is the primary interface with the aqueous phase and dictates much of the biosorption behaviour [38,39,41]. Different algal taxa have distinctly structured walls that preferentially interact with phenolic compounds via various chemical mechanisms [92]. In many microalgae and cyanobacteria, the wall contains abundant polysaccharides (cellulose, hemicellulose, pectins, or exopolysaccharides) rich in hydroxyl and carboxyl groups [93]. These polar functional groups readily form hydrogen bonds and dipole–dipole interactions with phenolic –OH groups [94,95]. For example, the outer sheath of *L. platensis* contains sulphated polysaccharides [96] as well as acidic uronic acids [97], which greatly increase surface polarity and water structuring; the many –OSO_3_^−^ and –COOH sites can engage phenolic oxygens via hydrogen bonding or even cation-mediated bridging if divalent ions are present.

By contrast, some green algae possess a trilaminar wall with an algaenan-rich outer sheath [98,99]. Algaenan and other long-chain aliphatic biopolymers impart hydrophobic character that favours van der Waals interactions and π–π stacking with aromatic rings [100,101]. While these hydrophobic domains can enhance adsorption of nonpolar organics, they can also sterically shield inner hydrophilic layers, limiting hydrogen-bonding interactions with polar solutes such as phenols. Diatoms add another modality because the silica frustule provides silanol (Si–OH) groups [91,102] and a large specific surface area; this inorganic scaffold offers strong hydrogen-bond donors/acceptors in addition to organic coatings that further diversify binding modes. The combination of inorganic and organic binding domains can make diatoms particularly effective in certain contexts [103,104], but it produces a sorption fingerprint distinct from cellulose- or peptidoglycan-dominated walls.

*L. platensis* is notable because its Gram-negative-type envelope (peptidoglycan, lipopolysaccharide and an amorphous polysaccharide sheath) exposes a chemically diverse and accessible surface rich in hydroxyl, carboxyl, amino and sulphate functionalities [88,105,106]. Unlike taxa encased in inert algaenan shields, *L. platensis*’ sheath is less blocking and presents many accessible groups for multi-point interactions with phenolic molecules [89,107]. This structural accessibility, combined with the presence of proteinaceous and aromatic residues on the outer envelope, explains the comparatively high phenol affinity commonly observed for *L. platensis* biomass in mixed-phenolic systems.

At the level of adsorption modelling, the heterogeneous distribution of site types and binding energies on algal walls often manifests as Freundlich-like behaviour. Indeed, our data fit the Freundlich isotherm better than Langmuir, consistent with a surface that offers a continuum of site affinities rather than a finite set of identical monolayer sites. The Freundlich exponent values and the absence of a clear saturation plateau indicate that high-affinity sites dominate at low equilibrium concentrations while additional uptake at higher concentrations is progressively less efficient [108]. This multi-site, mixed-mechanism reality has been reported in other algal sorption studies and aligns with the chemical diversity present on algal surfaces [108].

From a sustainability and process-design perspective, while adsorption kinetics (k) varied among species, most reached an adsorption plateau within approximately 10 min. This suggests that while speed is a relevant operational factor for reducing hydraulic retention times, the equilibrium capacity (q_e_) remains the more decisive parameter for long-term sustainability. A faster kinetic rate, such as that observed in *P. purpureum*, is advantageous but must be balanced against total capacity; a higher q_e_ directly reduces the environmental burden by requiring a lower total biomass dose to treat specific effluent volumes, thereby optimizing resource use throughout the cultivation and functionalization stages.

The selection of *L. platensis* for detailed investigation is therefore justified not only by its chemical performance but primarily by its superior biological productivity and industrial scalability. In industrial setups, *L. platensis* can achieve significantly higher biomass concentrations, reaching up to 5 g·L^−1^ [109], compared to other taxa like the diatom *C. weissflogii*, which typically reaches 0.5 to 1.0 g·L^−1^ [109,110]. This high productivity drastically reduces the environmental footprint per kilogram of biosorbent by minimizing the land, water, and energy required for agitation and harvesting, factors that represent the primary greenhouse gas drivers identified in our LCA (Figure 4). Consequently, *L. platensis* acts as a more efficient “biological factory”, facilitating its integration into cascading biorefinery systems where established market value and low production costs further improve the overall sustainability balance. This synergy between cell-wall chemistry and biomass yield demonstrates that the selection of a biosorbent cannot be based on adsorption capacity alone but must result from a holistic evaluation of the species’ industrial scalability. In this framework, a robust and accessible cell-wall architecture is the mechanistic prerequisite for effective phenol binding, but it is the superior biological productivity that ensures this performance translates into a net environmental and economic benefit for large-scale wastewater treatment.

#### Losses of Adsorption Capacity in *L. platensis* After Macromolecular Extraction

Our experimental observation that pigment/protein extraction abolishes phenol uptake in *L. platensis* underscores a fundamental mechanistic insight: adsorption is an emergent property of the intact cell-envelope matrix rather than the product of a single polymer class. During functionalization with HCl, some internal components of the cell, including pigments, may be exposed, and their extraction prior to functionalization reduces the potential for adsorption. If extraction occurs before functionalization, the biomass loses access to these internal components that could contribute to adsorption, including pigments, proteins, and polysaccharides. This suggests that the interaction between phenols and the biomass is dependent on the structural integrity of the full cell-envelope matrix, which includes both surface and internal components exposed or modified during the functionalization process.

Proteins, pigments, and polysaccharides form a three-dimensional, amphipathic scaffold that co-presents polar and nonpolar motifs in geometries favourable to multi-point adsorption [111,112]. Phycobiliproteins and other chromophoric complexes contain aromatic amino acids and conjugated systems that can engage in π–π interactions with phenolic rings [113,114], while protein side-chains supply amine and amide groups for hydrogen bonding [115]. Polysaccharides offer dense –OH and –COOH patterns for H-bonding and cation-mediated bridging [116,117]. Removing proteins and pigments by extraction thus eliminates both the functional groups and the structural microenvironments that stabilize phenol binding.

The results of our study differ from those reported by other researchers, who observed that algae used in biorefineries were still capable of adsorbing pollutants and heavy metals from the culture medium, sometimes even in greater amounts than non-extracted biomass [46,47,48]. However, most of these studies focus on lipid extraction rather than the macromolecules considered here, namely phycobiliproteins and proteins, which constitute the main products of *A. spirulina* in terms of both quantity and economic value. Some studies, such as Michalak et al. [118], confirm that the carboxylic groups of proteins and phycobiliproteins are primarily responsible for the adsorption of molecules onto the *L. platensis* cell wall, providing support for our observations. Nonetheless, data from the literature suggest a more nuanced picture, showing that the impact of extraction is highly specific to the pollutant, the extracted component, and the algal species. For example, a study investigating the removal of a model azo dye by *L. platensis* residue after phycocyanin extraction found that the dye biosorption capacity was largely retained [119]. The residual biomass exhibited a capacity of 23.06 mg g^−1^, very close to the 25.46 mg g^−1^ capacity of the untreated, virgin alga. This finding suggests that the binding of this particular dye may rely more on interactions with the remaining polysaccharide matrix, rather than on the protein–pigment complexes required for phenol binding.

Mechanistically, the loss of adsorption capacity can be explained by several concurrent effects. First, the absolute density of active functional groups on the surface decreases when proteins and pigments are removed [111]. Second, protein–polysaccharide crosslinks that maintain gel-like matrices and binding pockets collapse, reducing the accessible surface area for adsorption. Third, chelated metal ions associated with protein residues, often acting as cationic bridges, are lost during extraction [120,121]. Collectively, these changes reduce the number of sites, and the remaining matrix acts largely as a low-affinity polysaccharide surface.

These mechanistic insights have practical implications. For applications that aim to valorise algal biomass (for pigments, proteins or other bioactive) while also using the residue as an adsorbent, extraction protocols must be designed to preserve the cell-wall architecture and key functionalities. Mild extraction methods, targeted stabilization (e.g., crosslinking), or immobilization strategies that retain protein–polysaccharide assemblies could preserve absorptive performance. Conversely, if extraction is performed before adsorption, then the residue should not be assumed to be an effective biosorbent. Understanding the balance between bioproduct recovery and retention of adsorption capacity is therefore essential in any integrated algal biorefinery concept.

### 4.2. Limnospira Platensis as Biosorbent (Capacity and LCA Outcomes)

The equilibrium and kinetic behaviour of *L. platensis* reflect its chemically heterogeneous surface and the practical trade-offs of biosorption-based remediation. Our Freundlich fits indicate favourable uptake at low equilibrium concentrations, a desirable trait when the target is trace organic contaminants in effluents [122]. The observed uptake magnitudes (tens of mg of phenol per gram of biosorbent under our conditions) place *L. platensis* within the range reported for effective algal biosorbents, though not necessarily at the capacity extremes achieved by engineered activated carbons [123].

This capacity-versus-sustainability trade-off is where the LCA results are instructive. Although activated carbon typically achieves higher single-cycle removal performance, its production is energy- and chemical-intensive. High-temperature carbonization and chemical activation (with KOH or similar agents) generate substantial greenhouse gas emissions and resource consumption [69,124]. In our comparative LCA, the maize-derived activated carbon scenario accumulated a Global Warming Potential (GWP) an order of magnitude higher per functional unit than the *Spirulina* biosorption scenario (≈15 kg CO_2_eq/kg for activated carbon versus values as low as ≈3 kg CO_2_eq/kg for *L. platensis*, Figure 4). Electricity for furnace heating and the embodied impacts of KOH activation were the dominant contributors to the activated carbon route’s GWP. By contrast, *Spirulina* cultivation requires nutrients and some energy, but photosynthetic carbon fixation offsets a portion of upstream emissions, making the overall GWP per unit of phenol removed markedly lower.

From an application perspective, these results suggest different niches for each approach. Activated carbon remains attractive for applications where very high instantaneous removal is required, but its environmental cost must be considered. *L. platensis*-based biosorption is compelling for low-to-moderate removal targets where life cycle sustainability and co-product potential are prioritized. In particular, when the phenolic contaminants are natural compounds, such as those from olive mill wastewater (OMWW) used in our study (and in Mollo et al. [54]), the spent *Spirulina* biomass may have enhanced value. This potential is supported by studies showing that natural phenolics act as effective antioxidants in feed supplements [125,126,127], combining the established value of *L. platensis* as an additive [128] with the beneficial properties of the adsorbed compounds [50]. Such synergy can be leveraged in a biorefinery concept, improving the overall sustainability balance [129]. Nonetheless, in this LCA (Figure 1, Table 5), it was decided to adopt a conservative boundary by modelling the end-of-life for both the algal biomass and activated carbon as hazardous waste treatment. Even under this “worst-case” disposal scenario, the algal route remains environmentally superior, demonstrating that its benefits are robust regardless of whether the biomass is recycled or disposed of.

Moreover, the potential use of live *L. platensis* deserves explicit consideration. Living cultures add active removal mechanisms, biodegradation and metabolic assimilation, on top of passive biosorption. Several studies, including Lee et al. [130], demonstrate that for phenol, biodegradation by living cells is the predominant and more effective removal mechanism compared to the reversible biosorption by dead biomass. Live remediation, however, imposes stricter operational constraints and complicates downstream biomass handling, as it may be unsuitable for food or feed applications.

Operational strategies can bridge the performance gap between *Spirulina* and activated carbon while retaining sustainability benefits. Such strategies include mild physicochemical modifications to increase the density of functional groups [123] or treating the spent biomass as a feedstock for secondary processes, such as conversion to biochar [131], thereby closing a material loop. This thermochemical valorisation, rather than chemical desorption, is the preferred management strategy as it effectively stabilizes the organic pollutants and recovers energy without generating secondary liquid waste streams, directly addressing the environmental implications of handling loaded biosorbents.

### 4.3. Future Perspectives

The results of this study point to several clear priorities for future research. First, process optimization: Improving uptake capacity through mild modifications, hybrid materials, or immobilization [126,127] could make *L. platensis* more competitive with activated carbon on a per-mass basis. Second, integration with biorefinery concepts: Coupling remediation with the recovery of valuable fractions (e.g., phycobiliproteins) can significantly improve economic and environmental outcomes [12]. Post-extraction biomass residues retain value as protein- and antioxidant-rich food ingredients, closing the loop in high-value applications [50].

Third, scaling and operational design require attention. Regarding operational feasibility, the deployment of fine algal powder necessitates a downstream separation step to prevent biomass carryover into the treated effluent. This challenge is analogous to that faced when using Powdered Activated Carbon (PAC) and can be addressed through standard industrial unit operations, such as coagulation–flocculation followed by sedimentation or the integration of membrane filtration (e.g., membrane bioreactors). To validate these integrated configurations, future pilot deployments should test continuous flow reactors and regeneration strategies. The potential to cultivate *L. platensis* on nutrient-rich secondary streams (e.g., agricultural wastewaters) offers a path toward system circularity [129].

Fourth, exploring live-alga remediation and controlled biotransformation pathways is promising [130,131]. Genetic or adaptive approaches could enhance phenol tolerance, increase expression of degradative enzymes, or favour sequestration pathways that promote irreversible removal. But any such approach must be carefully evaluated for biosafety and for downstream product implications.

Finally, regulatory, market, and life cycle considerations should not be neglected. If biomass containing adsorbed phenolics is destined for food or feed chains, strict safety assessments are necessary. Policy incentives and circular-economy frameworks could favour algae-based remediation where co-products are recognized and regulated appropriately [132].

## 5. Conclusions

This study identifies the presence, type, and biochemical integrity of the cell wall as the decisive factors for phenolic biosorption. Our screening revealed that while wall-less species showed negligible uptake, cyanobacteria exhibited the highest removal efficiency, outperforming other taxa. Crucially, experiments on *Limnospira platensis* demonstrated that the extraction of proteins or pigments completely abolished adsorption. This proves that the mechanism relies on the cooperative architecture of an intact cell wall matrix; consequently, chemically altered residues are ineffective for this application.

Despite this limitation on extracted residues, the whole *L. platensis* biomass proves to be an environmentally superior alternative to commercial activated carbon. The Life Cycle Assessment (LCA) confirms that the algal route drastically reduces the Global Warming Potential by avoiding the energy-intensive carbonization and chemical activation steps required for conventional adsorbents.

Therefore, the optimal implementation strategy for a circular economy is not the pre-extraction of metabolites, which compromises performance, but the valorisation of the post-sorption biomass. Transforming the “spent” biomass (e.g., via conversion to biochar) ensures that the remediation efficiency is maximized while still closing the resource loop.

## Figures and Tables

**Table 1:** Microalgal and cyanobacterial species screened in this study. Species names (with strain identifiers where available), taxonomic group, culture origin and cell-wall type are reported.

Phylum	Habitat	Algal Species	ID Code	Type of Cell Wall
Chlorophyta	Freshwater	*Tetradesmus obliquus*	CCAP 276/3A	Cellulose and algaenan
	Freshwater	*Chlorella vulgaris*	CCAP 211/11b	Cellulose and algaenan
	Marine	*Dunaliella salina*	Isolated from Margherita di Savoia saltpans (Italy)	No cell wall
Rhodophyta	Marine	*Porphyridium purpureum*	CCAP 1380/3	No rigid cell wall, sulphated polysaccharides
Haptophyte	Marine	*Isochrysis galbana*	Roscoff RCC 1353	No rigid cell wall, organic scales
Bacillariophyte	Marine	*Conticribra weissflogii*	DCG 0320	Siliceous frustule
	Marine	*Phaeodactylum tricornutum*	UTEX 646	Siliceous frustule
Ochrophyte	Marine	*Nannochloropsis salina*	CCAP 849/3	Polysaccharides and algaenan
Cyanobacteria	Marine	*Synechococcus* sp.	UTEX LB 2380	Peptidoglycan
	Freshwater	*Anabaena* sp.	CCAP 1403/4a	Peptidoglycan
	Freshwater	*Limnospira platensis*	SAG 85.79	Peptidoglycan

**Table 2:** Coefficient of phenol adsorption by different species (initial phenol 30 mg L^−1^, biomass 0.1 g L^−1^). Quantity of phenol adsorbed at equilibrium (q_e_) and pseudo first-order rate constant of biosorption (k) are reported as mean ± standard deviation. Letters represent significant differences between species.

Species	Phylum/Type	q_e_ (mg·g^−1^)				k (ms^−1^)			
*Tetradesmus obliquus*	Chlorophyta	67.7	±	8.1	ac	6.0	±	2.0	a
*Chlorella vulgaris*	Chlorophyta	12.3	±	1.4	d	2.7		1.5	a
*Dunaliella salina*	Chlorophyta	<LOD				n.a			
*Porphyridium purpureum*	Rodophyta	42.3	±	26.4	acd	18.3	±	2.9	a
*Isochrysis galbana*	Haptophyta	<LOD				n.a			
*Conticribra weissflogii*	Bacillariophyta	56.5	±	13.6	ac	16.0	±	7.8	a
*Phaeodactylum tricornutum*	Bacillariophyta	39.6	±	12.3	bcd	13.7	±	11.7	a
*Nannochloropsis salina*	Ochrophyte	30.3	±	8.1	cd	2.7	±	0.6	a
*Synechococcus* sp.	Cyanobacteria	74.2	±	25.8	ba	2.3	±	0.6	a
*Anabaena* sp.	Cyanobacteria	81.5	±	7.8	a	12.7	±	4.9	a
*Limnospira platensis*	Cyanobacteria	78.7	±	4.2	a	11.3	±	7.6	a

**Table 3:** Equilibrium adsorption results of HCl-functionalized Limnospira platensis biomass at different doses (0.1–1.0 g·L^−1^) in a 30 mg·L^−1^ phenolic solution. Values are reported as mean ± standard deviation (*n* = 3).

Algal Dose (g L^−1^)	C_e_ (mg L^−1^)			q_e_ (mg·g^−1^)			Removal (%)		
0.10	22.3	±	0.6	76.9	±	6.0	25.7	±	2.0
0.20	19.3	±	0.7	53.3	±	3.6	35.5	±	2.4
0.33	13.8	±	0.6	48.7	±	1.8	54.1	±	2.0
1.00	11.8	±	0.3	18.3	±	0.3	60.8	±	1.0

**Table 5:** Life cycle inventory for the two assessed scenarios: (1) *L. platensis* biomass as biosorbent and (2) maize-based activated carbon. Reported values include energy inputs, chemical consumption, and operational conditions considered in the LCA analysis, and they referred to a functional unit of 1 L of phenolic solution. Maize production was accounted for using an emission factor of 0.0575 kgCO_2_/kg; therefore, while the specific inventory data for cultivation are omitted from this table, the resulting environmental impact is fully integrated into the LCA analysis.

SCENARIO 1					
INPUT			OUTPUT		
Algal Growth					
NaHCO_3_	15.1	g	Spent growth medium with A. platensis biomass	220	g
K_2_HPO_4_	0.4	g			
NaNO_3_	2.25	g			
K_2_SO_4_	0.91	g			
NaCl	0.91	g			
MgSO_4_ × 7H_2_O	0.18	g			
CaCl_2_	0.04	g			
FeSO_4_ × 7H_2_O	0.01	g			
EDTA	0.08	g			
A5 solution	0.01	g			
Led light	5.04	kWh			
Distilled H_2_O	220	g			
Autoclave	0.094	KWh			
**Centrifugation**					
Energy	0.14	KWh	*A. platensis* biomass	1	g
H_2_O for balancing	220	g	Spent growth medium	220	g
Spent growth medium with *A. platensis* biomass	220	g	H_2_O	220	g
**Washing and centrifugation**					
*A. platensis* biomass	1	g	Washed algal biomass	1	g
H_2_O for biomass washing	440	g	H_2_O	660	g
H_2_O for balancing	220	g			
Energy	0.14	kWh			
**Functionalisation**					
Washed algal biomass	1	g	Functionalised algal biomass	1	g
HCI 35%	0.09	g	Wastewater rich in HCI	10	g
H_2_O for dilution	9.91	g	H_2_O	10	g
Energy	0.185	kWh			
H_2_O for balancing	10	g			
**Dehydration**					
Functionalised algal biomass	1	g	Dry algal biomass	1	g
Energy	0.072	kWh			
**Adsorption and mixing**					
Energy	0.003	kWh	Remediated wastewater	1	L
Phenolic solution	1	L	Biomass rich in phenols	1	g
Dry algal biomass	1	g			
**Wastewater treatment**					
Spent growth medium	220	g	Discharged H_2_O	1.14	kg
H_2_O	918	g			
Wastewater rich in HCI	0.09	g			
**Waste treatment**					
Biomass rich in phenols	1	g	Treated biomass	1	g
**SCENARIO 2**					
**INPUT**			**OUTPUT**		
**Grinding**					
Energy	0.011	kWh	Maize powder	0.25	g
Maize	0.25	g			
**Dehydration**					
Maize powder	0.25	g	Dehydrated powder	0.25	g
Energy	5.88	kWh			
**Carbonisation**					
Energy	0.22	kWh	Carbonized powder	0.25	g
Nitrogen	37.5	g			
Dehydrated powder	0.25	g			
**Mixing**					
Biomass	0.25	g	Mixture	1.25	kg
Potassium hydroxide	1	kg			
**Activation**					
Mixture	1.25	kg	Activated carbon	1.25	kg
Nitrogen	37.5	g			
Energy	13	kWh			
**Washing**					
H_2_O	1	L	Wet carbon		
Activated carbon	1.25	kg			
**Vacuum drying**					
Energy	10.8	kWh	H_2_O	2	L
Wet Carbon	2.25	kg	Dry activated carbon	0.25	g
**Adsorption and mixing**					
Phenolic solution	1	L	Remediated wastewater	1	L
Dry activated carbon	0.25	g	Activated carbon rich in phenols	0.25	g
Energy	0.03	kWh			
**Wastewater treatment**					
H_2_O	2	L	Discharged H_2_O	2	L
**Waste treatment**					
Activated carbon rich in phenols	0.25	g	Treated carbon	0.25	g

## Data Availability

Data will be available on reasonable request.
